# The Role of Structural Dynamics of Actin in Class-Specific Myosin Motility

**DOI:** 10.1371/journal.pone.0126262

**Published:** 2015-05-06

**Authors:** Taro Q. P. Noguchi, Masatoshi Morimatsu, Atsuko H. Iwane, Toshio Yanagida, Taro Q. P. Uyeda

**Affiliations:** 1 Biomedical Research Institute, National Institute of Advanced Industrial Science and Technology, Tsukuba, Ibaraki, Japan; 2 Graduate School of Life and Environmental Sciences, University of Tsukuba, Ibaraki, Japan; 3 Department of Chemical Science and Engineering, National Institute of Technology, Miyakonojo College, Miyakonojo, Miyazaki, Japan; 4 Nanobiology Laboratories, Graduate School of Frontier Biosciences, Osaka University, Suita, Osaka, Japan; 5 Quantitative Biology Center (QBiC), RIKEN, Suita, Osaka, Japan; 6 Graduate School of Medical Life Science, Yokohama City University, Tsurumi, Yokohama, Kanagawa, Japan; Semmelweis University, HUNGARY

## Abstract

The structural dynamics of actin, including the tilting motion between the small and large domains, are essential for proper interactions with actin-binding proteins. Gly146 is situated at the hinge between the two domains, and we previously showed that a G146V mutation leads to severe motility defects in skeletal myosin but has no effect on motility of myosin V. The present study tested the hypothesis that G146V mutation impaired rotation between the two domains, leading to such functional defects. First, our study showed that depolymerization of G146V filaments was slower than that of wild-type filaments. This result is consistent with the distinction of structural states of G146V filaments from those of the wild type, considering the recent report that stabilization of actin filaments involves rotation of the two domains. Next, we measured intramolecular FRET efficiencies between two fluorophores in the two domains with or without skeletal muscle heavy meromyosin or the heavy meromyosin equivalent of myosin V in the presence of ATP. Single-molecule FRET measurements showed that the conformations of actin subunits of control and G146V actin filaments were different in the presence of skeletal muscle heavy meromyosin. This altered conformation of G146V subunits may lead to motility defects in myosin II. In contrast, distributions of FRET efficiencies of control and G146V subunits were similar in the presence of myosin V, consistent with the lack of motility defects in G146V actin with myosin V. The distribution of FRET efficiencies in the presence of myosin V was different from that in the presence of skeletal muscle heavy meromyosin, implying that the roles of actin conformation in myosin motility depend on the type of myosin.

## Introduction

The actin molecule consists of two major domains (the small and large domains) separated by a deep cleft that binds ATP or ADP. The cleft has been demonstrated to open or close depending on the nucleotide state [1–3]. This conformational change regulates the binding of certain actin-binding proteins. For example, profilin has lower affinity for the ADP—actin monomer than for the ATP—actin monomer [4,5] and binds astride the small and large domains. Thus, profilin may distinguish conformational differences between these two major domains according to the nucleotide state. The relative positions of the two major domains of the monomeric form and of the subunits within filaments are also different. Accompanying polymerization, the two major domains rotate in a propeller-like manner, with the actin subunits adopting a flatter conformation. Oda *et al*. (2009) suggested that this flat conformation contributes to the stability of filaments [6]. Nonetheless, actin subunits in filaments do not always take one state [7–10]. The conformational variation of actin subunits within a filament may play an important role in the regulation of interactions with various actin-binding proteins in the cells.

Propeller rotation within the actin subunit to the flat structure occurs with a certain lag after incorporation into filaments [8]. Before the rotation, the two domains in the actin subunits are tilted relative to each other, and the actin subunits in the twisted conformation dissociate from the filaments more readily than does the flat form (discussed in a review [11]). Interestingly, among the possible conformational states of actin subunits in filaments [12], one state is stabilized by cofilin. The state stabilized by cofilin has two twisted major domains, disrupting contacts with neighboring subunits within a filament [13]. This characteristic of cofilin may contribute to its function in severing actin filaments. Conformational changes in actin filaments may also be involved in myosin motility, since modifications of actin filaments by proteolytic cleavage or chemical cross-linking impairs the motility of myosin without significantly affecting the binding affinity and the ability to stimulate myosin ATPase activity [14–16]. These results indicate that the flexibility of or conformational changes in actin filaments are important for force generation by myosin. However, conformational states of actin subunits induced by or appropriate for interaction with myosin are not well understood.

Transitions between different conformational states of actin subunits in a filament have been hypothesized to occur cooperatively. For example, depolymerization of a single actin filament proceeds at one rate during release of a few thousand actin subunits and then switches to a different rate [17]. Furthermore, cofilin binds cooperatively to actin filaments [12,18–20], involving cooperative conformational changes in the filaments (described in a review [21]). Cofilin-induced cooperative conformational changes in actin subunits accompany significant supertwisting of the helical pitch, which propagates to neighboring subunits [22]. Myosin also induces cooperative conformational changes in actin filaments [10,23–25] and binds cooperatively to actin filaments [26,27]. Thus, myosin-induced cooperative conformational changes in actin filaments may also involve propeller rotation between the two major domains.

The absolutely conserved Gly146 (in the *Dictyostelium* Act15 sequence without the removed first Met) of actin sits at the hinge between the two domains. We previously reported that the G146V mutation of actin dominantly inhibits the growth of yeast cells [28] and impairs the *in vitro* gliding motility by skeletal myosin II but not of myosin V [29]. We speculated that the G146V mutation somehow affects the propeller rotation between the two major domains, which in turn impairs the motility of myosin II but not that of myosin V. In the present study, we performed filament depolymerization assays and single-molecule intramolecular FRET measurements to test this hypothesis. We used a newly developed FRET-probed actin with donor and acceptor fluorophores attached to the tips of the two domains.

## Materials and Methods

### Protein preparation

Skeletal heavy meromyosin (sk MII HMM) and the heavy meromyosin equivalent of cytoplasmic myosin V (MV HMM) were prepared as described previously [30,31]. Mutant *Dictyostelium act15* genes for FRET analysis, T41Q/S239C/C374A and T41Q/G146V/S239C/C374A, were generated by PCR-based methods. Wild-type (WT) and mutant actins were purified as described previously [32]. G146V and WT actins for the depolymerization assays were labeled with N-(1-pyrene)iodoacetamide (Invitrogen, Tokyo, Japan), as described previously [33].

Biotin-labeled actin was prepared as follows. Solutions of polymerized WT or G146V actin dissolved in 10 mM HEPES pH 7.4, 2 mM MgCl_2_, 100 mM KCl, and fivefold molar excess of biotin—PEAC5–maleimide (Dojin, Kumamoto, Japan) were mixed and incubated for 2 hr at room temperature. The reaction was stopped by addition of 5 mM DTT. Labeled F-actin was ultracentrifuged at 300,000×g for 15 min at room temperature, and the resultant pellet was dissolved and dialyzed against G-buffer (2 mM Tris-HCl pH 7.4, 0.2 mM CaCl_2_, 0.2 mM ATP, 0.5 mM DTT). The supernatant after ultracentrifugation was used as biotin-labeled actin.

### Polymerization and depolymerization assays

Before polymerization, actin (20%, pyrene-labeled) in G-buffer was supplemented with 1 mM EGTA and 50 μM MgCl_2_ to convert Ca—actin to Mg—actin and then incubated on ice for 10 min [34]. Polymerization was induced by adding 100 mM KCl, 2 mM MgCl_2_, and 0.5 mM ATP. After polymerization for 12 min or overnight at 22°C, 30 μM latrunculin A (Sigma, Tokyo, Japan) was added to induce depolymerization. Rates of polymerization and depolymerization were monitored by measuring the fluorescence intensity of pyrene.

The critical concentrations of WT and G146V actins were measured by sedimentation assays. Different concentrations of actin (0.5 μM~3.0 μM) were polymerized in F-buffer (10 mM HEPES pH 7.4, 2 mM MgCl_2_, 100 mM KCl, 0.5 mM ATP, 0.5 mM DTT) for 3 hr at 21°C, and were ultracentrifuged. Amounts of protein in pellets were determined by Advanced Protein Assay Reagent (Cytoskeleton, Denver, CO).

### Labeling of actin with FRET probes

Purified T41Q/S239C/C374A and T41Q/G146V/S239C/C374A mutant actins were dialyzed against LG-buffer (5 mM Tris-HCl pH 8.0, 1 mM CaCl_2_, 0.4 mM ATP, 0.01% NaN_3_) and then diluted to 30 μM with the same buffer. One-twentieth volume of 30 mM Alexa Fluor 647 C2 maleimide (Invitrogen) dissolved in DMSO was added to these actin solutions, and the mixtures were incubated on ice overnight. The reaction was stopped by adding 3 mM DTT. Actins labeled with Alexa Fluor 647 were further labeled with Alexa Fluor 555 cadaverine (Invitrogen) via a transglutaminase reaction using a modification of a reported method [35]. In brief, Alexa Fluor 647 labeled actin, 150 μM Alexa Fluor 555 cadaverine and 0.3 unit/ml transglutaminase (Sigma) were combined, and the resulting mixture was incubated overnight at 4°C. The labeled actins were polymerized by adding 1/10 vol of 10×F-buffer (100 mM HEPES pH 7.4, 20 mM MgCl_2_, 1 M KCl). After incubation for 1 hr at room temperature, they were ultracentrifuged at 300,000×g for 15 min. The resultant pellets were dissolved and dialyzed against G-buffer and then re-ultracentrifuged. The doubly labeled mutant actins were rapidly frozen and stored in liquid nitrogen. The concentration of doubly labeled actin was measured from its band density in SDS-PAGE using skeletal actin of known concentration as standard. Concentrations of Alexa Fluor 555 and Alexa Fluor 647 were measured by using the extinction coefficients of each fluorescent dye (Alexa Fluor 555: 150,000 M^-1^ cm^-1^ at 555 nm; Alexa Fluor 647: 239,000 M^-1^ cm^-1^ at 650 nm) (Invitrogen). The Förster distance of this FRET pair is 5.1 nm (Invitrogen, http://www.lifetechnologies.com/jp/ja/home/references/molecular-probes-the-handbook/tables/r0-values-for-some-alexa-fluor-dyes.html).

### FRET measurement system

Control or G146V FRET actin was copolymerized with the corresponding unlabeled and biotin-labeled WT or G146V actins at a molar ratio of 10:100:1 in buffer at room temperature for 1 hr. The buffer contained 20 mM HEPES (pH 7.8), 5 mM MgCl_2_, 25 mM KCl, 1 mM EGTA, 0.5 mM DTT, 0.5 mM ATP, and 1.2-fold molar excess of phalloidin (Sigma). Phalloidin has no effect on the motility defect of sk MII HMM with G146V mutation [29].

Two coverslips of different sizes and double-sided adhesive tape were used to construct a flow cell. To immobilize the FRET actin filaments, 2 mg/ml BSA containing 0.2 mg/ml biotinylated BSA (Sigma) was introduced into the flow cell, which was then incubated for 2 min. Unbound BSA was washed away with FRET buffer (20 mM HEPES pH 7.8, 1 mM EGTA, 25 mM KCl, 5 mM MgCl_2_). Subsequently, 1 mg/ml streptavidin was added, and the cell was incubated for 4 min. After extensive washing with FRET buffer, FRET actin filaments were introduced into the flow cell, which was then incubated for 5 min and then washed with FRET buffer. Finally, M-buffer (20 mM HEPES pH 7.8, 1 mM EGTA, 25 mM KCl, 5 mM MgCl_2_, 100 mM DTT, 1 mM ATP, oxygen scavenger system [36], and ATP regeneration system [37]) containing either 2.5 μM sk MII HMM or 30 nM MV HMM and 0.2 mg/ml calmodulin was introduced.

FRET was monitored by using a custom-made total internal reflection fluorescence microscope [37] equipped with a 100 X objective lens. The donor was excited by a diode laser at 532 nm. Fluorescence of the donor and acceptor was accumulated for 0.1 s and stored successively by using an electron multiplying CCD camera (Ixon, Andor Technologies, Belfast, UK).

### Data analysis

Fluorescence spots, which had fluorophores bleached in one step, were used in the calculation of FRET efficiency (E). Light intensities after both fluorophores were bleached (background) were subtracted from the fluorescence intensities of donor and acceptor, and the FRET efficiency was calculated by using the expression *I*_*a*_/(*I*_*d*_•γ + *I*_*a*_). The γ-value, which is a correction factor for fluorescence, accounts for the ratio of donor and acceptor quantum yields and detection efficiencies. We adopted the γ-value from our previous report [37], which used the same fluorescent dyes and FRET measurement system. Individual data series of FRET efficiency were then smoothed by using a moving average of five frames. Fluorescence spots, of which one-third of smoothed FRET efficiencies were over 1.0, were excluded from further analysis. The resultant data series of FRET efficiencies from multiple fluorescence spots were combined to construct a distribution histogram. Each FRET efficiency distribution was then fitted with Gaussian models through the Mclust package in the R software (R Development Core Team, 2005, website: www.r-project.org). Optimal fits were selected through the Bayesian information criterion (BIC) that determines the best parameterization of the model and the optimal number of clusters based on the likelihood of the data [38]. In a separate analysis, we calculated the means of the FRET efficiencies to compare overall FRET efficiencies under different experimental conditions.

## Results and Discussion

### Polymerization and depolymerization properties of G146V actin

**Fig 1:**
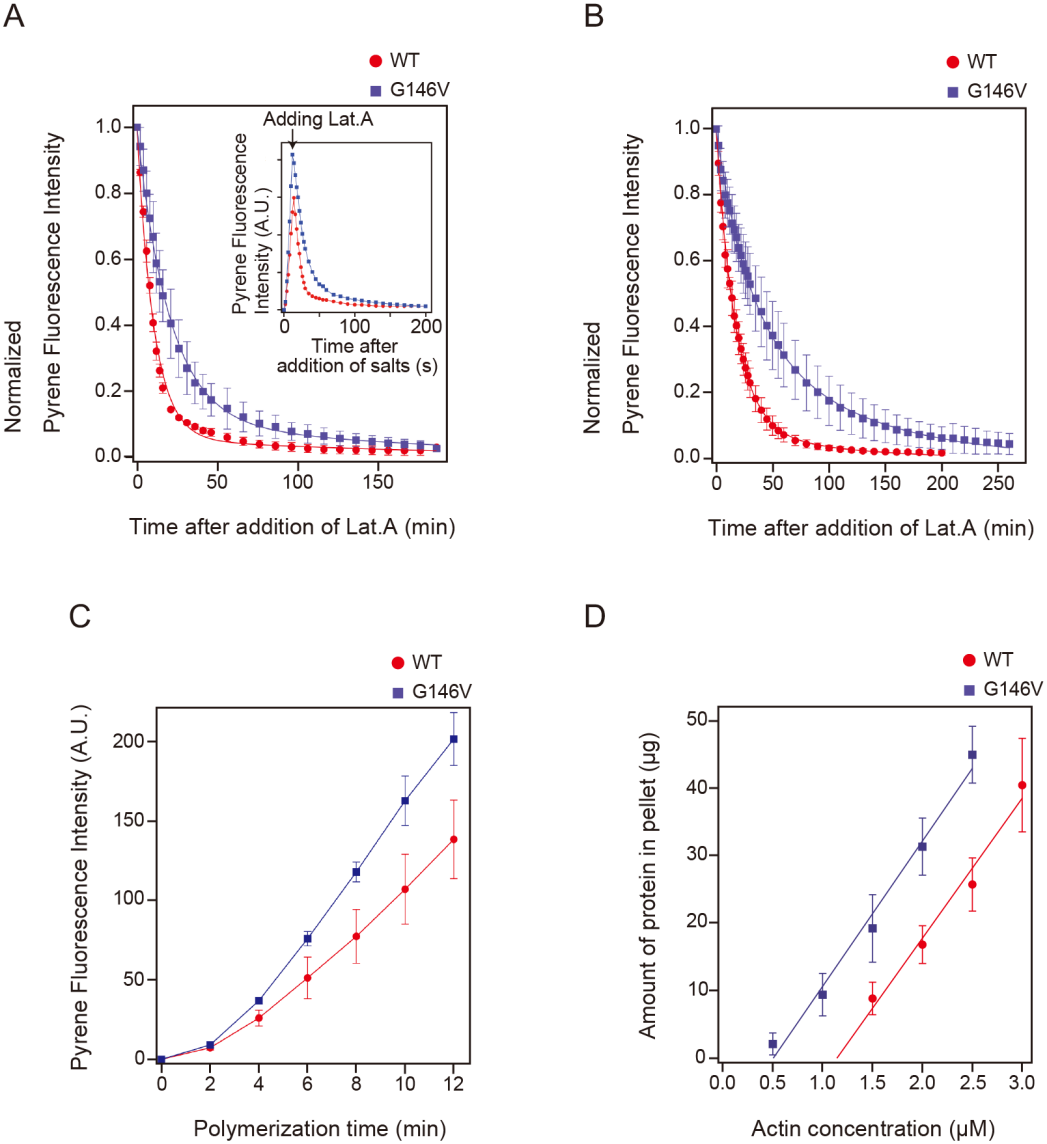
Polymerization and depolymerization of G146V and WT filaments. A 2 μM solution of monomeric actin (20% labeled with pyrene) was polymerized for 12 min (A) or overnight (B) at 22°C. Depolymerization was then induced by adding 30 μM latrunculin A. Data were fitted with a double-exponential equation, *I(t)* = *f*sexp(-t/τs) + (1 − *f*s)exp(-t/τf) [17]. The parameters *f*s and τs respectively give the fraction and depolymerization rate of the slow-depolymerization population, and τf gives the depolymerization rate of the fast-depolymerization population. Inset shows the raw data before normalization, including those for the polymerization phase before addition of latrunculin A. (C) Trace of polymerization before addition of latrunculin A. Error bars indicate standard deviation (n = 3). (D) The critical concentrations of G146V and WT actins were measured by pelleting assay. Actins of 0.5~3.0 μM were polymerized for 3 hr, and were ultracentrifuged. Amounts of protein in resultant pellets were determined by Advanced Protein Assay Reagent. Error bars indicate standard deviation (n = 3).

Depolymerization rate of actin filaments varies depending on polymerization time, which is believed to derive from delayed relative rotation between the two domains after incorporation into filaments [11]. Thus, *Dictyostelium* actin filaments polymerized for 12 min or overnight were depolymerized by adding excess latrunculin A (Fig 1). The depolymerization rate of G146V filaments was significantly slower than that of WT filaments, regardless of the incubation periods (Fig 1A and 1B). Depolymerization rates of both WT and G146V filaments polymerized for 12 min were higher than those for overnight incubation. As depolymerization process consists of two components [17], we fitted each set of depolymerization data with a double-exponential equation (Table 1). Overall, the lower depolymerization rate of the G146V filaments was due to a larger fraction of slowly depolymerizing component. After aging, the slow component of WT filaments increased about twofold, while that of the G146V filaments increased about fourfold. On the other hand, depolymerization rates of the slow component of WT and G146V filaments did not differ significantly. These results suggest that the G146V subunits favor the conformational state similar to the slowly depolymerizing state of the WT filaments. Thus, G146V mutation may stabilize one of the native states of actin subunits. A global conformational change in the actin molecule involving rotation between the small and large domains has been implicated in stabilization of filaments [6]. In the flat conformation, the hydrogen side-chain of Gly146 is oriented inward [39]. The environment around this hydrogen is hydrophobic, surrounded by side-chains of 142Leu, 152Val, 298Val and 330Ile. Modeling suggested that the introduced Val residue at the 146^th^ position fits in this space without steric clash and interacts with these hydrophobic residues (S1 Fig). In contrast, in the tilted state, the hydrogen side-chain of Gly146 is oriented outward, so that the side-chain of Val residue at the 146^th^ position would be exposed to the surrounding water in this conformation. Thus it is plausible that the flat conformation of G146V actin is more stable than the tilted conformation, due to the hydrophobic interaction, resulting abundance of the slowly depolymerizing fraction in G146V filaments. The lower critical concentration of G146V actin (Fig 1D, WT actin vs G146V actin = 1.1 μM vs 0.51 μM) also suggests that G146V actin is predisposed to take the flat conformation. It should be noted, however, that the hydrogen side-chain of Gly 146 is pointing outward in other structures of actin in the flat conformation [6,40].

**Table 1:** Parameters of depolymerization.

Parameters	12 min		Overnight	
	WT	G146V	WT	G146V
ƒ_*s*_	0.06 ± 0.02	0.12 ± 0.031	0.11 ± 0.03	0.47 ± 0.05
τ_*s*_ (min)	184 ± 120	169 ± 61	87 ± 19	98 ± 7
τ_*f*_ (min)	10.3 ± 0.4	20.1 ± 0.85	16.7 ± 0.5	27.4 ± 2.0

Parameters were obtained by fitting data in Fig 1A and 1B with double exponentials. The parameters ƒ_*s*_ and τ_*s*_ respectively give the fraction and depolymerization rate of the slow-depolymerization population, and τ_*f*_ gives the depolymerization rate of the fast-depolymerization population. Mean and standard deviation of three independent measurements are shown.

The polymerization rate of G146V actin during the initial 12 min incubation was higher than that of WT actin (Fig 1C). This is consistent with the rapid change in G146V subunits to the flat conformation immediately upon their incorporation into filaments, which stabilizes the filament structure. Alternatively, monomeric G146V actin may tend to take a conformation that is incorporated into the filaments more readily than the WT monomers. This latter possibility is consistent with the result of our previous study, which showed that monomeric actin under polymerization conditions shift the conformational equilibrium to a polymerization-competent intermediate state [37].

### Importance of structural dynamics of actin to myosin motility

Cryo-electron microscopy (cryo-EM) studies and ensemble biophysical measurements demonstrated that binding of certain actin-binding proteins changes the structure of actin subunits, suggesting that the functions of actin-binding proteins rely on the dynamics of actin structure. However, analysis of the dynamics of actin structure has been difficult because cryo-EM observation requires freezing of actin filaments, and ensemble measurements average out heterogeneous structural states. Thus, we used single-molecule intramolecular FRET measurement to observe the dynamic behavior of actin structures during myosin motility.

**Fig 2:**
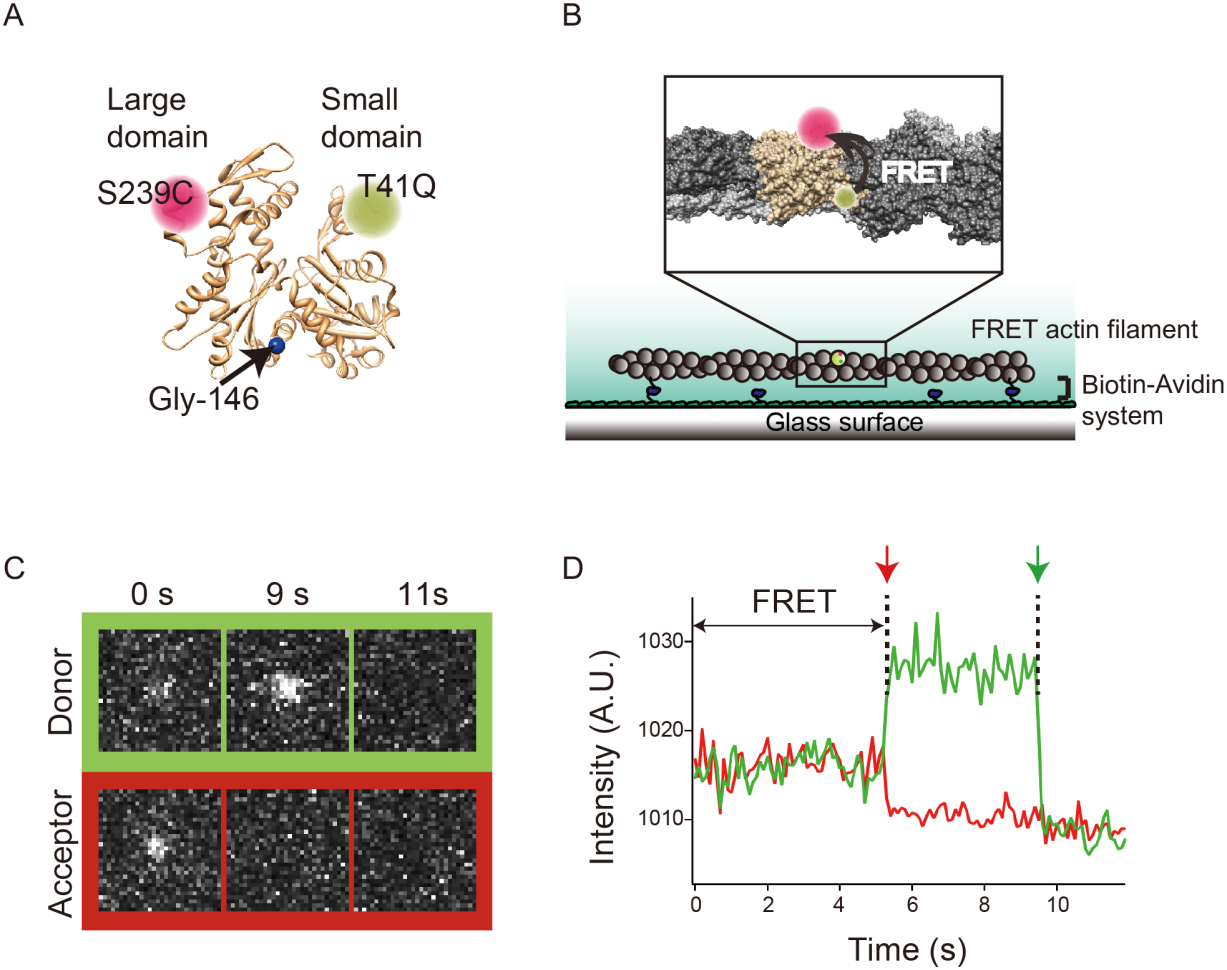
Intramolecular FRET measurement system. (A) Donor and acceptor fluorophores were introduced at the tips of the two major domains of WT actin and G146V actin (FRET actin). Actin monomer (PDB ID: 1c0f) [48] and filament (PDB ID: 3g37) [41] were drawn by using UCSF Chimera software. (B) FRET actins were copolymerized with excess amounts of WT or G146V actin, 0.9% of which was biotinylated, and they were immobilized on a glass surface by using the biotin—avidin system. (C and D) A typical series of time-lapse fluorescence images and fluorescence intensities of donor and acceptor from a single actin subunit in a filament in the absence of myosin. During FRET, fluorescence of the acceptor (red) was higher than that of the background, while that of the donor (green) was lower. Upon photobleaching of the acceptor (red arrow), the fluorescence intensity of the donor increased. Subsequently, the donor was also bleached (green arrow).

By resolving the distribution of FRET efficiencies of individual actin molecules to multiple Gaussian distributions, Kozuka et al. [9] and Morimatsu et al. [37] revealed that actin molecules take two or more different structural states. These studies demonstrated the unique usefulness of this approach in the detection of the structural dynamics of actin subunits. However, these studies introduced both acceptor and donor fluorophores into the small domain. This approach is inappropriate for detecting propeller rotation between the small and large domains that the G146V mutation is expected to affect. Thus, we selected the 41st and the 239th residues of actin for fluorescent labeling, because these residues are located near the tips of the small and large domains, respectively (Fig 2A), and because the side-chains of these two residues are oriented outward in the monomeric form [41] and are therefore accessible from the solvent. Furthermore, labeling of Gln41 in skeletal actin with dansyl cadaverine does not affect interaction with myosin II [35], and Ser239 is not located in the binding site for myosin [42].

On the basis of this design, WT and G146V *Dictyostelium* actin molecules were engineered to carry Gln and Cys residues in place of Thr41 and Ser239, respectively. WT actin has four Cys residues, but only one of them (Cys374) is highly reactive [43]. Thus, we replaced Cys374 with Ala to produce T41Q/S239C/C374A and T41Q/G146V/S239C/C374A mutant actins. These actins were sequentially labeled with Alexa Fluor 647–maleimide and Alexa Fluor 555–cadaverine. Typical labeling efficiencies with Alexa Fluor 647–maleimide and Alexa Fluor 555–cadaverine were approximately 80% and 20%, respectively. These double-labeled actins are hereafter referred to as control FRET actin and G146V FRET actin, respectively. The FRET actins were copolymerized with unlabeled and biotin-labeled WT or G146V actins at a molar ratio of 10:100:1, and they were stabilized with phalloidin. These filaments were then subjected to single-molecule intramolecular FRET assays. All experiments were performed in the presence of 1 mM ATP.

**Fig 3:**
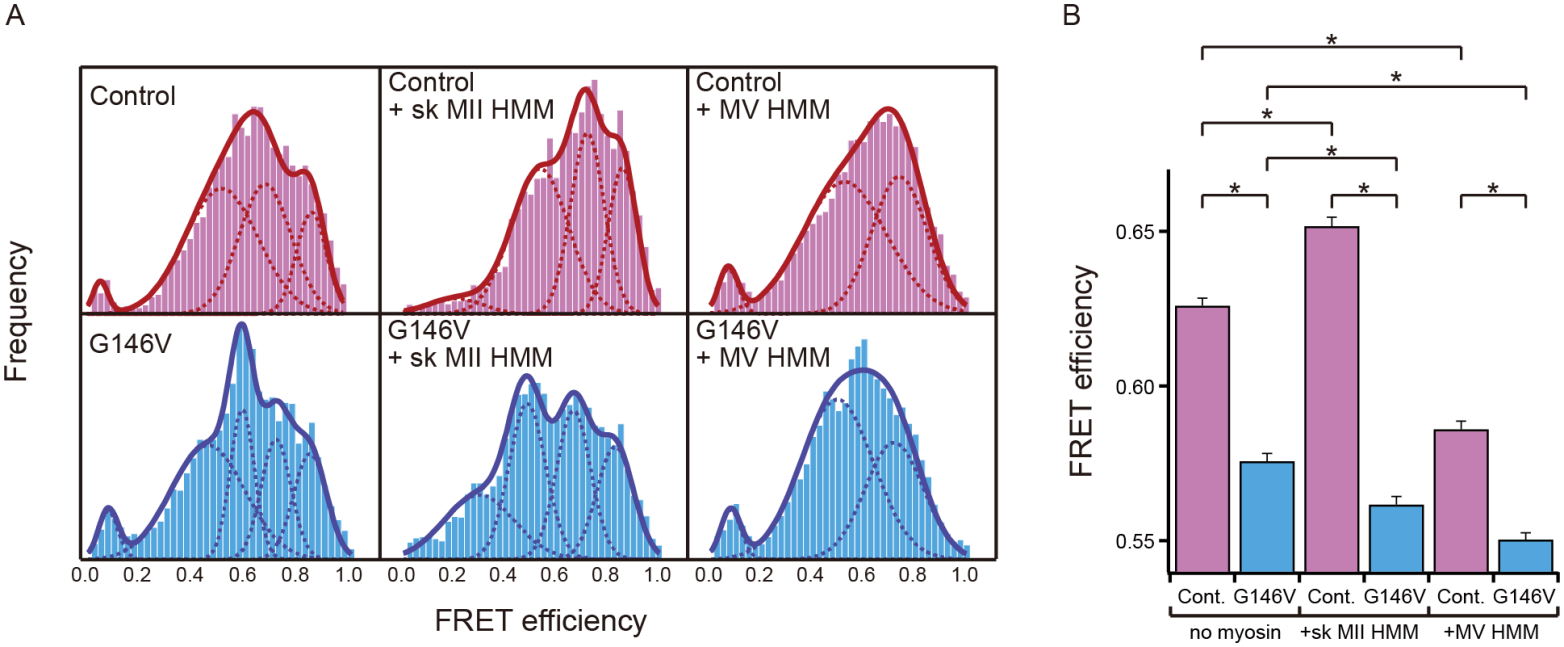
FRET distributions. (A) Upper and lower panels show histograms of the FRET efficiencies of control FRET actin (red) and G146V FRET actin (blue), respectively. Assays were performed in the absence (left) and in the presence of sk MII HMM (middle) or MV HMM (right). The dashed lines and thick lines show each component of the fitted Gaussian distributions and their sum, respectively. The number of data points subjected to FRET analysis is shown in S1 Table. (B) Changes in mean values calculated from overall FRET efficiencies induced by interaction with HMM. Statistical significance was assessed through the Mann—Whitney U-test. Asterisks indicate statistical significance (p < 0.00001). Error bars show SEM. All assays were performed in the presence of 1 mM ATP and the ATP regeneration system.

We observed fluorescence from single actin subunits within filaments, as evidenced by bleaching of each fluorophore in one step (Fig 2C and 2D). In this case, we observed inverse relationships between donor and acceptor emission intensities, as well as transition between distinct FRET states (S2 Fig). Each histogram of FRET efficiencies, combined from 63–102 fluorescent spots in the absence or presence of either myosin, was fitted with multiple Gaussian distributions. The appropriate number of Gaussian distributions was determined from the BIC for each histogram. All of the FRET distributions were well fitted with the sum of three to five Gaussian models (Fig 3A). These results suggest that both WT subunits and G146V subunits took multiple distinct conformational states, each of which had a unique distance between tips of the two major domains. However, we could not determine whether resolving the distributions to four or five distinct populations, suggested as appropriate by the BIC, is meaningful.

Mean values of each Gaussian distribution of FRET efficiencies of control FRET subunits in the absence of myosin were 0.049, 0.52, 0.69, and 0.88 (Table 2). The distances between fluorophores in these FRET states, as calculated from the equation R = R_0_(1/E − 1)^1/6^ under the assumption of random orientations of the two fluorophores, were 8.4, 5.0, 4.5, and 3.7 nm. Distance of 8.4 nm is much larger than the size of an actin molecule (~5 nm), which means that this minor fraction of FRET represents inter-molecular FRET. Results of a recent electron microscopic analysis of skeletal actin filaments suggest that there are at least six different conformations [7]. The similarity of distances between Cys239 and Gln41 among some of the six conformations may explain why we saw less than six distinct Gaussian distributions in the control FRET efficiencies.

**Table 2:** Parameters of the FRET efficiencies obtained by Gaussian fitting.

	None		sk MII HMM		MV HMM	
	Control	G146V	Control	G146V	Control	G146V
Number of Gaussian distributions	4	5	4	4	3	3
mean ± variance *probability* of FRET efficiencies	0.049 ± 0030	0.077 ± 0.038	0.206 ± 0.108	0.296 ± 0.135	0.069 ± 0.119	0.078 ± 0.040
	*0*.*025*	*0*.*048*	*0*.*044*	*0*.*231*	*0*.*045*	*0*.*052*
	0.523 ± 0.144	0.456 ± 0.140	0.536 ± 0.111	0.485 ± 0.072	0.523 ± 0.165	0.494 ± 0.143
	*0*.*471*	*0*.*418*	*0*.*424*	*0*.*294*	*0*.*569*	*0*.*598*
	0.694 ± 0.101	0.586 ± 0.046	0.723 ± 0.066	0.666 ± 0.067	0.739 ± 0.108	0.717 ± 0.114
	*0*.*346*	*0*.*18*	*0*.*313*	*0*.*264*	*0*.*386*	*0*.*35*
	0.875 ± 0.059	0.716 ± 0.057	0.861 ± 0.057	0.828 ± 0.071		
	*0*.*158*	*0*.*178*	*0*.*22*	*0*.*212*		
		0.844 ± 0.064				
		*0*.*178*				

These parameters were obtained by fitting data in Fig 3 with Gaussian distributions.

To interpret the FRET data in a fitting-independent manner, we compared mean values of overall FRET efficiencies in each experimental condition. The mean value of FRET efficiency of G146V FRET actin in the absence of myosin was significantly lower than that of control FRET actin (Fig 3B). This conformational difference should be correlated with the altered polymerization properties (Fig 1). However, the lower overall FRET efficiencies is opposite to what is expected from mutant actin’s slower depolymerization rate and lower critical concentration, and hence abundance of the flat conformation. Furthermore, they are not consistent with the prediction of the structural modeling (S1 Fig). We suggest that this is presumably related to the orientation of the fluorophores, which also affects FRET efficiencies. This is because Gln41 can be labeled with Alexa 555 Fluor cadaverine when actin is in the monomeric form but not when in the filament form [35], suggesting the possibility that the Alexa Fluor 555 fluorophore attached to Gln41 is confined in a narrow space and its movement is restricted when polymerized.

The difference in FRET distributions between control and G146V FRET actins in the presence of 2.5 μM sk MII HMM was significantly larger than in the absence (Fig 3A). A lower FRET state of G146V FRET actin, which had a mean FRET efficiency of 0.30 (Table 2), appeared. We observed this state with control FRET actin as well, but its population was much smaller than that of G146V FRET actin. Furthermore, changes in FRET distribution induced by sk MII HMM were different between control and G146V FRET actins. Mean of FRET efficiencies of control FRET actin was increased by sk MII HMM, while that of G146V FRET actin was decreased (Fig 3B). These results suggest that an increase in the higher FRET state(s) is necessary for the motility of myosin II. The minor, very low FRET distribution in the control FRET actin disappeared, suggesting that myosin II also affects relationships between actin subunits.

MV HMM decreased the FRET efficiencies of both the control and G146V FRET actins (Fig 3A). Means of FRET efficiencies of control and G146V FRET actins were different, but the difference was smaller than that in the presence of sk MII (Fig 3B).

### Difference between requirements for actin conformation associated with motilities of myosins II and V

It is well established that during the ATPase cycle, actin and myosin alternate between the strongly bound state and the weakly bound or detached state. Furthermore, the strongly bound state consists of several chemical states, including A•M•ADP•Pi, A•M•ADP, and A•M states [44]. Each actin subunit may undergo a cyclic conformational change concurrently with this mechanochemical cycle. However, the duration of the strongly bound state is on the order of milliseconds in the presence of saturating concentrations of ATP [45], and it does not occupy a significant fraction of the 100 ms accumulation time to acquire one fluorescence image. Therefore, one may argue that even if the strongly bound state or its substate has a higher FRET efficiency, this hypothetical high FRET state is unlikely to change the FRET intensity averaged over 100 ms and thus unlikely to affect the distribution of the observed FRET efficiencies. This conclusion is consistent with the fact that very few of the observed fluorescent spots cyclically alternated between two distinct FRET efficiencies in the presence of sk MII HMM.

However, the conformational change in an actin subunit induced by strong binding of a single sk MII HMM molecule might affect multiple neighboring actin subunits in the filaments in a cooperative manner [10,23–25]. If so, our FRET measurement might be able to detect the conformational changes induced by strongly bound sk MII HMM when the sk MII HMM concentration was sufficiently high. It is also possible that, once transient strong interaction with a single sk MII HMM molecule had changed the conformation of an actin subunit, the new conformation persisted after the HMM molecule had dissociated. This scenario is consistent with our earlier observation that spontaneous conformational switching of actin subunits was slow [37]. In either case, effects on the filament may be detectable even if the duration of each strong-binding event was very short.

In contrast, the duration of the weakly bound state may be on the order of several hundred milliseconds under our conditions. This would be long enough to cover several consecutive data points and would affect the distribution of FRET intensities if the weakly bound crossbridges increase the fraction of a particular conformation of actin. The preferred conformation may be either a new conformation absent in the control FRET actin without MII HMM or one of the four distinct distributions observed.

One can interpret these latter possibilities from a different point of view, assuming that actin had at least two functionally distinct conformations in relation to interaction with sk MII HMM. One conformation is an off or refractory state, which binds only slowly to sk MII HMM carrying ADP and Pi, and/or does not produce large displacement and force even after it binds to sk MII HMM. The other conformation is an on state, which interacts with sk MII HMM and produces force and displacement efficiently. In this scenario, the on state has a higher FRET efficiency than the off state, and the G146V mutant actin does not easily take the on state, either kinetically or thermodynamically. Consistent with this hypothesis, our earlier biochemical study suggests that the motility defect of G146V actin filaments with myosin II derives from two defects; i.e., slower transition from the weakly bound state to the strongly bound state and the lower affinity during the strongly bound state [29].

The interaction of G146V actin filaments with myosin V produced normal velocity and force [29], even though mean values of the FRET efficiencies during interaction with MV HMM were somewhat different between control and G146V actins (Fig 3B). Similarly, Kubota et al. reported that M47A mutation of actin halved both gliding velocity and force due to myosin II, while it increased these parameters due to myosin V [46]. These results suggest the possibility that the mechanism for motility is qualitatively different between processive myosin V (reviewed in [47]) and non-processive myosin II, and accordingly, the roles of actin filaments in the motilities of myosin II and V are not identical. In this case, the conformation of actin filaments impaired by the G146V mutation is important only in the motility of myosin II. This view is consistent with a study by Prochniewicz et al. on transient phosphorescence anisotropy [10], which showed that conformational states of actin induced by myosins II and V are different. However, an alternative, more conservative explanation is also possible. Because myosin V motor domains remain bound to actin filaments much longer than myosin II motor domains do, they may have the larger potential to cause the off state G146V actin subunits to switch to the on state. Nonetheless, it should be noted that conformational changes induced by MII and MV were distinct (Fig 3A). Further studies are needed for a better understanding of the roles that actin conformations play in myosin motilities.

## Supporting Information

S1 Fig Environment around Val 146. Gly146 of actin in the flat (left, PDB ID: 3j8i) and the tilted (right, PDB ID: 3j8j) conformation was substituted by Val using UCSF Chimera software. Red and green spheres indicate carbon atoms in Val146 and those in hydrophilic residues around Val 146, respectively.
(TIF)

S2 Fig Transition of FRET. Selected traces of the fluorescence intensities of donor (green) and acceptor (red) before subtraction of the background, and of the FRET efficiency (magenta) of control FRET actin. Left, middle, and right traces were obtained in the absence of myosin, in the presence of sk MII HMM, and in the presence of MV HMM, respectively.
(TIF)

S1 Table Number of data points subjected to FRET analysis. (DOCX)
