# Supplementary figures and images for: The Role of Structural Dynamics of Actin in Class-Specific Myosin Motility
Source: PLoS One. 2015 May 6;10(5):e0126262. doi: 10.1371/journal.pone.0126262 (PMC4422724; doi:10.1371/journal.pone.0126262)

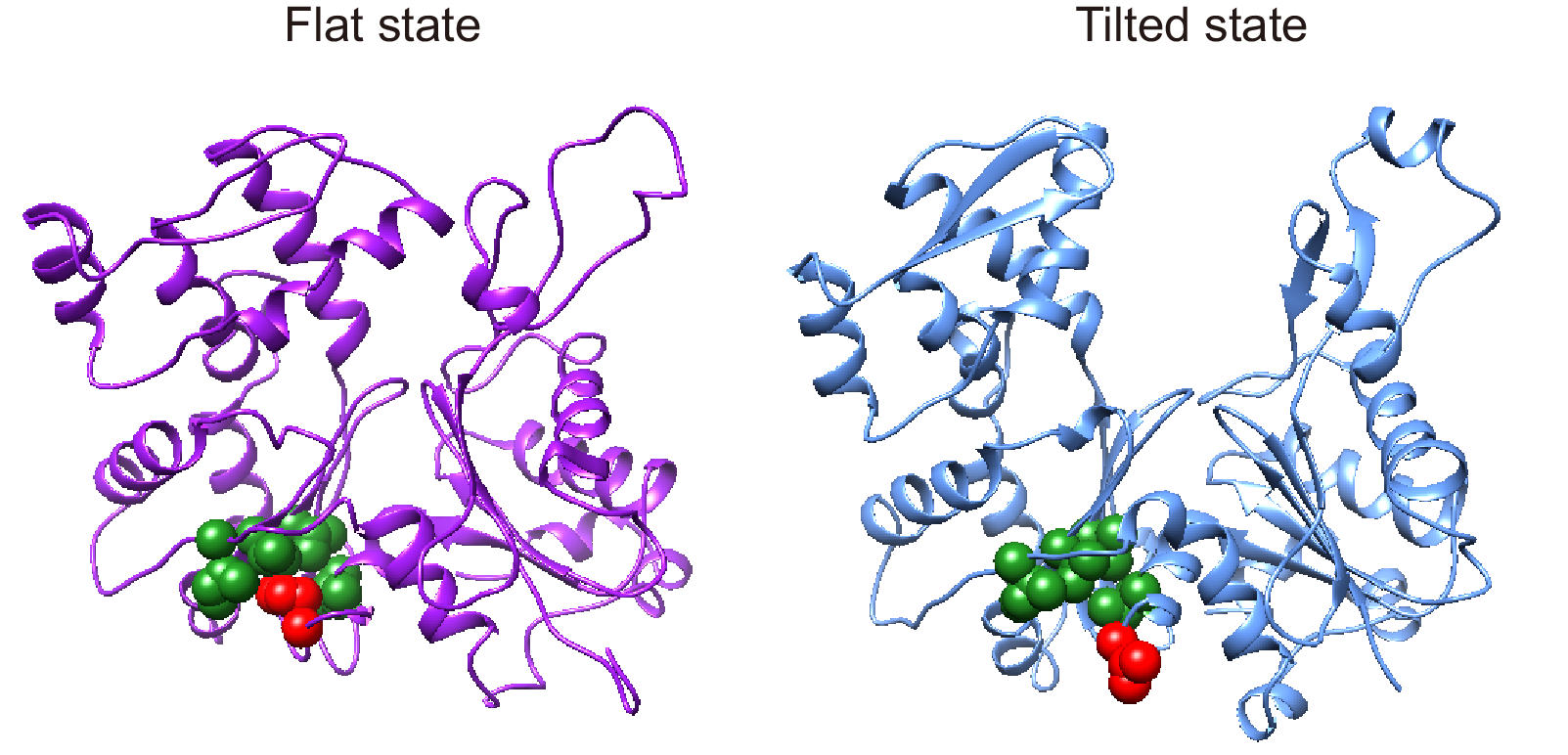

Supplement: S1 Fig — Gly146 of actin in the flat (left, PDB ID: 3j8i) and the tilted (right, PDB ID: 3j8j) conformation was substituted by Val using UCSF Chimera software. Red and green spheres indicate carbon atoms in Val146 and those in hydrophilic residues around Val 146, respectively. (TIF) [file pone.0126262.s001.tif]

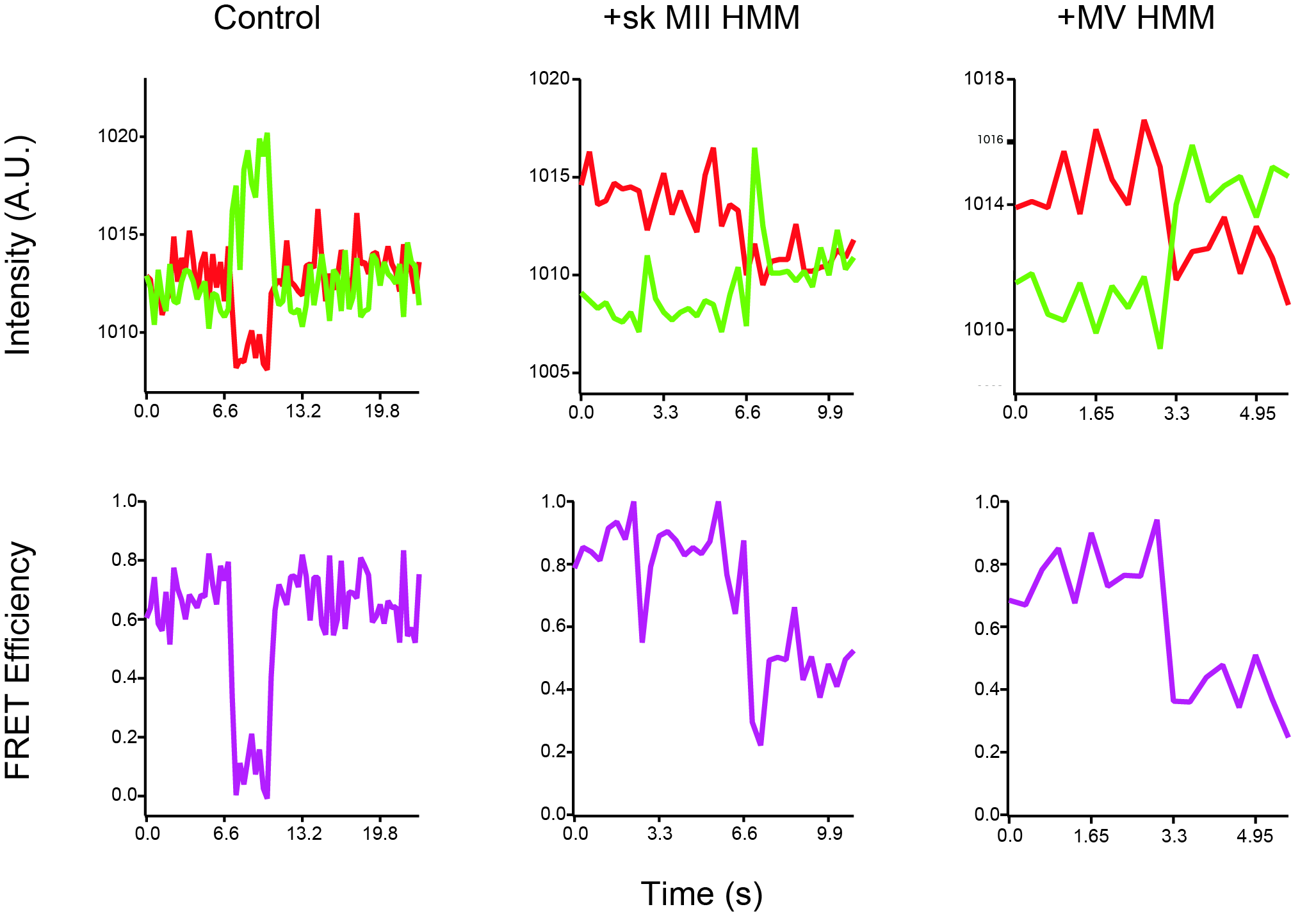

Supplement: S2 Fig — Selected traces of the fluorescence intensities of donor (green) and acceptor (red) before subtraction of the background, and of the FRET efficiency (magenta) of control FRET actin. Left, middle, and right traces were obtained in the absence of myosin, in the presence of sk MII HMM, and in the presence of MV HMM, respectively. (TIF) [file pone.0126262.s002.tif]
