# Supplementary material for: The Role of Structural Dynamics of Actin in Class-Specific Myosin Motility
Source: PLoS One. 2015 May 6;10(5):e0126262. doi: 10.1371/journal.pone.0126262 (PMC4422724; doi:10.1371/journal.pone.0126262)
Supplement: S1 Table — (DOCX) [file pone.0126262.s003.docx]

|  | no myosin | | sk MII HMM | | MV HMM | |
| --- | --- | --- | --- | --- | --- | --- |
|  | Control | G146V | Control | G146V | Control | G146V |
| Number of  fluorescence spots | 96 | 89 | 63 | 77 | 91 | 102 |
| Number of data points | 6536 | 6913 | 4063 | 6362 | 6411 | 8879 |

**Table S1. Number of data points subjected to FRET analysis**
